# Manipulation of Ovarian Function Significantly Influenced Trabecular and Cortical Bone Volume, Architecture and Density in Mice at Death

**DOI:** 10.1371/journal.pone.0145821

**Published:** 2015-12-30

**Authors:** Jeffrey B. Mason, Boston C. Terry, Samer S. Merchant, Holly M. Mason, Mahdi Nazokkarmaher

**Affiliations:** 1 Department of Animal, Dairy and Veterinary Sciences, Center for Integrated BioSystems, School of Veterinary Medicine, Utah State University, Logan, UT, United States of America; 2 Department of Bioengineering, College of Engineering, University of Utah, Salt Lake City, UT, United States of America; Nanjing Medical University, CHINA

## Abstract

Previously, transplantation of ovaries from young, cycling mice into old, postreproductive-age mice increased life span and decreased cardiomyopathy at death. We anticipated that the same factors that increased life span and decreased cardiomyopathy could also influence the progression of orthopedic disease. At 11 months of age, prepubertally ovariectomized and ovary-intact mice (including reproductively cycling and acyclic mice) received new 60-day-old ovaries. At death, epiphyseal bone in the proximal tibia and the distal femur and mid-shaft tibial and femoral diaphyseal bone was analyzed with micro-computed tomography. For qualitative analysis of osteophytosis, we also included mineralized connective tissue within the stifle joint. Prepubertal ovariectomy had the greatest influence on bone volume, ovarian transplantation had the greatest influence on bone architecture and both treatments influenced bone density. Ovarian transplantation increased cortical, but not trabecular bone density and tended to increase osteophytosis and heterotopic mineralization, except in acyclic recipients. These effects may have been dictated by the timing of the treatments, with ovariectomy appearing to influence early development and ovarian transplantation limited to influencing only the postreproductive period. However, major differences observed between cycling, acyclic and ovariectomized recipients of new ovaries may have been, in part due to differences in the levels of hormone receptors present and the responsiveness of specific bone processes to hormone signaling. Changes that resulted from these treatments may represent a compensatory response to normal age-associated, negative, orthopedic changes. Alternatively, differences between treatments may simply be the 'preservation' of unblemished orthopedic conditions, prior to the influence of negative, age-associated effects. These findings may suggest that in women, tailoring hormone replacement therapy to the patient's current reproductive status may improve therapy effectiveness and that beginning therapy earlier may help preserve trabecular bone mineral density that would otherwise be lost during perimenopause.

## Introduction

Osteoarthritis is often considered a compensatory response to joint instability and is influenced by the mechanisms controlling bone remodeling [1]. In the female, these remodeling mechanisms are strongly influenced by ovarian function and are significantly altered by ovarian aging and reproductive senescence [2]. Although rare in young women, orthopedic disease becomes increasingly prevalent during and after the menopausal transition. Menopausal arthritis is often considered a distinct form of arthritis, where the instigating cause is hormonal, not genetic or mechanically related [3]. It is characterized by an early, perimenopausal loss of trabecular bone mineral density (BMD). Postmenopausally, loss of trabecular BMD subsides and trabecular volume is maintained (albeit, at a lower level) in the absence of ovarian estrogen (E2) and progesterone (P4) [4,5]. However, bone quality is not determined solely by BMD. The microarchitecture of bone contributes significantly to bone strength and durability [6,7]. Bone architecture and bone densitometry are both subject to the effects of ovarian senescence.

A common, but consistently controversial course of action at the menopausal transition is to remedy disrupted ovarian signaling with exogenous hormones through hormone replacement therapy (HRT). The 'critical period' hypothesis suggests that there is a 'critical window' early, during perimenopause where HRT is effective, but that HRT loses its general effectiveness and may be detrimental if initiated later in the postmenopausal years [8,9]. The existence of the 'critical period' is hypothesized to result from long-term hormone deprivation, which leads to a decreased capability for E2 signaling. In rats, long-term ovarian hormone deprivation attenuated the ability of HRT to regulate levels of E2 receptors (ER), [10]. Both young and old mice with senescent ovaries are less responsive to exogenous E2 treatment [11].

The destruction of periarticular soft tissue in diarthrodial joints over the course of a life time is often so great that the condition of the joint cannot be assessed solely from the remaining soft tissue. However, bone persists and leaves a valuable measure of joint history at the time of death. In humans, collection of bone tissues for ex vivo analysis is often limited to samples taken during joint replacement or those collected at death (analysis often involves levels of radiation exposure incompatible with in vivo analysis). Additionally, there are few reports describing the skeletal changes that occur in the latter half of the mouse lifespan, which in the female would reflect age-associated changes in ovarian function.

Previously, we successfully modified ovarian hormone signaling in aged female mice by transplanting ovaries from young mice to old, postreproductive-aged (11 months of age) mice [12]. Half of the intact, postreproductive-aged transplant recipients were still showing some signs of reproductive cycling and were under the influence of actively-cycling, albeit aged ovaries. The other half had completely ceased cycling prior to the time of transplantation and therefore, experienced a lapse in cyclic ovarian influence prior to receiving new ovaries. Additional recipients had been prepubertally ovariectomized (OVX) and never experienced any cyclic ovarian input prior to receiving new ovaries at 11 months of age. The transplantation of young ovaries into postreproductive-age mice increased life span [12], decreased the rate of unintentional weight loss at advanced ages [13] and decreased cardiomyopathy at death [14]. We predicted that the same factors in transplant recipients that increased life span and decreased cardiomyopathy could also decrease orthopedic disease progression. In the current paper, we report the results of the micro-computed tomography (μCT) analysis of bones in mice with and without the influence of active ovaries at different stages of the life span. We include quantitative analysis of the proximal and mid-shaft tibia and the distal and mid-shaft femur. For qualitative analysis of osteophytosis, we also include mineralized connective tissue within the stifle joint. Significant differences in bone volume, architecture and density were detected due to prepubertal OVX and ovarian transplantation and between mice that were reproductively cycling and mice that had ceased cycling at the time of transplantation of new ovaries.

## Material and Methods

### Mice

The CBA/J strain (used in the current study) and the DBA strain of mice are unique in that they prematurely lose their ovarian follicles, becoming reproductively senescent by 10–12 months of age [15,16,17]. A reduction of ovarian follicles in the human is associated with the onset of menopause. For this reason, CBA/J mice may serve as an appropriate experimental model to study age-related changes in the human [18,19].

Adult breeders (40g) CBA/J strain female mice (Jackson Laboratory, Bar Harbor, ME) were provided ad libitum access to feed (LabDiet 5008: LabDiet, St. Louis, MO 63144; http://www.labdiet.com/cs/groups/lolweb/@labdiet/documents/web_content/mdrf/mdi4/~edisp/ducm04_028444.pdf) and water (deionized) and were housed under conditions of constant temperature (21°C ± 2°C), humidity (min. 50%), and lighting (14L: 10D, lights-on at 0700 h). Individual pups were weaned and ear-notched at 21 days (day of birth = 0 days). All female weanlings were housed individually, with added enrichment, in a 26 x 17 x 13 cm shoebox cage in a specific-pathogen-free colony where pathology on sentinel mice was done quarterly and pathological results showed no breach in this status. Mice were maintained in an American Association for Accreditation of Laboratory Animal Care (AAALAC)-approved facility in accordance with the National Institutes of Health animal-use guidelines. Animal care and use protocols were developed under National Research Council guidelines found in the Guide for the Care and Use of Laboratory Animals. This project was approved by the University of California, Davis Institutional Animal Care and Use Committee.

Anesthetics were used during surgery and analgesia was provided for 48 hours post-surgery, longer if deemed necessary. Mice were euthanized by asphyxiation with carbon dioxide, supplied via compressed gas cylinder. Animals with acute, but not severe weight loss were treated with subcutaneous fluids and moistened food. Animals with acute, but not severe urine staining or rectal/vaginal prolapse were manually cleaned and treated with Desitin. Mice were monitored at least twice daily and weights/photographs were recorded monthly, more frequently when concerns arose. Aged, moribund mice found with overt clinical signs were euthanized. Criteria for euthanasia included, but were not limited to mice found in poor condition with or without crusting around the anal area and diarrhea, urine staining, persistent vaginal prolapse, chronic vulva/rectal swelling, hunched posture, labored/agonal breathing, significantly decreased food intake, poor coat condition and lack of grooming, depression, hind-limb weakness/paresis, wounds not healing, limited mobility, obvious neoplastic growth and unusual weight loss (or gain). Average weight loss in aged mice, from peak weight to death in this experiment was approximately 12% per month. An increased the rate of weight loss, but not total weight loss was the most critical factor for determining a moribund state. Criteria for euthanasia specific for aged mice were determined in coordination with the attending veterinarian. Unexpected deaths were uncommon, but included neoplastic growths (most commonly mammary), unhealed wounds (extremely old animals) and uncontrolled seizures (normally between 11–13 months of age).

### Experimental design

**Fig 1:**
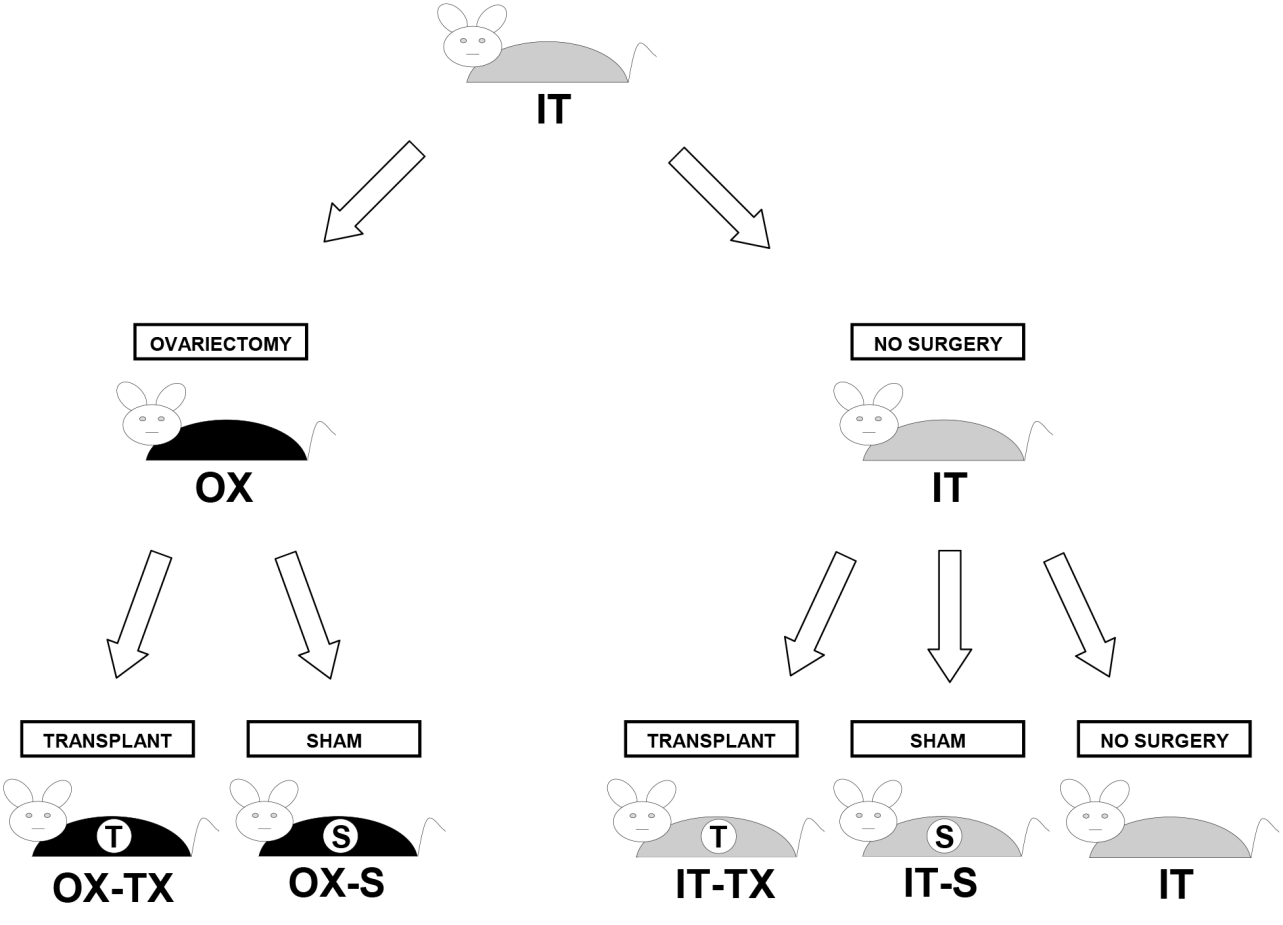
Experimental Design. IT (intact), OX (prepubertally OVX), S (sham surgery) and TX (ovarian transplantation). n = 10 for each μCT scanned group.

Animals were randomly assigned to sham or ovarian-transplant groups as follows (Fig 1):

**Shams consisted of**: IT-S: Intact sham animals remained intact to 11 months, at which time they underwent a sham surgery, which involved bilateral OVX and subsequent replacement of the endogenous ovaries back into their original bursae. OX-S: Ovariectomized sham animals were prepubertally OVX at 21 days and subsequently, at 11 months, underwent a sham surgery where we removed the 1-mm glass, place-holder beads, previously placed into the ovarian bursae during the 21-day prepubertal OVX procedure (see Surgical Procedures).

**Ovarian transplants consisted of**: IT-TX: Intact transplant animals remained intact to 11 months, at which time their endogenous ovaries were removed and replaced with a pair of donor ovaries from a 60-day-old mouse. For analysis, intact transplant mice were subdivided into mice that were still cycling at the time of transplantation (IT-TX-c) and mice that had ceased cycling or were acyclic at the time of transplantation (IT-TX-a). OX-TX: Ovariectomized transplant animals were prepubertally OVX at 21 days and subsequently, at 11 months, the 1-mm glass beads previously placed into the ovarian bursae during the 21-day OVX procedure were removed and replaced with a pair of donor ovaries from a 60-day-old mouse.

Sham treatments served as surgery controls for transplant procedures. All animals in each experimental group remained virgin until death.

### Age at manipulation

Mice of the CBA/J strain become reproductively competent between 45 and 60 days of age. Ovariectomy at 21 days of age was chosen to avoid major up-regulation of the reproductive system at the onset of puberty and to eliminate other influences the female gonad might have in addition to direct effects of gonadal hormones. These influences may include positive or negative feedback mechanisms or system-wide 'imprinting' influences that the active ovary may normally provide after 21 days of age. Rodents do not undergo menopause, but instead have an estropause-like decrease in reproductive function. Reproductive decline in CBA/J mice usually begins with irregular cycles at 8–10 months of age. At 11 months of age, many CBA/J mice have become reproductively incompetent [20] with a complete loss of oocytes by 12 months of age. Ovarian transplantation and sham surgeries were conducted at 11 months of age. Many females in this line of mice have reached a point of reproductive failure by this time with approximately half of these mice displaying complete lack of reproductive cycling and half of the mice continuing to cycle.

### Surgical procedures

Bilateral prepubertal OVX at 21 days and ovarian transplantation and sham surgeries at 11 months were performed as previously described [21]. Briefly, the ovaries were exposed by paralumbar incision under sodium pentobarbital anesthesia and removed by incising the ovarian bursa opposite the ovarian hilum. The ovary was gently removed from the ovarian bursa and excised by clamping the ovarian hilum to prevent bleeding. Excised ovaries were placed in cold saline prior to transfer/replacement. After transfer/replacement, the ovarian bursa was closed with one to three sutures of 10–0 Ethilon monofilament (Ethicon, Inc.). The abdominal wall was sutured with 5–0 chromic gut (Ethicon, Inc.), and the skin was closed with 9 mm wound clips (MikRon Precision, Inc.). When we performed ovariectomies at 21 days of age, a sterile 1-mm diameter glass bead was inserted into each empty ovarian bursa to keep it open for future ovarian transplantation or sham surgery.

At 11 months of age, bilateral ovarian transplantation and sham surgeries were performed as previously described [21]. Briefly, OX-S animals were subjected to sham ovarian transplant surgery in which the glass bead placed at 21 days of age was removed, but no ovary was transplanted. IT-S animals underwent sham ovarian transplant surgery in which their endogenous ovaries were removed, placed in cold saline and then returned to the original bursae.

IT-TX animals at 11 months of age underwent a bilateral OVX and subsequent ovarian transplantation and received a pair of 60-day-old ovaries from a donor mouse of the same strain. The OX-TX cohort underwent ovarian transplantation in which the glass beads placed at 21 days of age were removed and replaced with a pair of ovaries from a 60-day-old donor mouse of the same strain. Data on vaginal cytology were collected for at least 10 consecutive days pre- and post-surgery to ensure: 1) complete removal of the ovarian tissue, and 2) success of the ovarian transplantation procedure. Daily vaginal cytology was re-initiated beginning 10–14 days postoperatively. One estrous cycle was defined as the period from the day nucleated epithelial cells first appeared (i.e., proestrus) to the day preceding the next appearance of nucleated epithelial cells in the vaginal smear, provided there was a period of leukocytic presence (i.e., diestrus) in between. Estrus was determined by the presence of large, squamous epithelial cells, with or without nuclei. No immunosuppressive techniques were employed and no evidence of graft-versus-host disease was detected post-transplantation or at death. After surgery, each female was housed individually in a 26 x 17 x 13 cm shoebox cage. Additionally, all serum samples submitted from mice at the time of necropsy were negative for parvovirus.

### Exclusion criteria

Presumptive OVX mice that displayed cytological evidence of gonadal input prior to surgery at 11 months were excluded from analysis. Gonadal input was defined as cyclic changes on vaginal cytology, presumably due to cyclic influence of ovarian hormones. No gonadal input was defined as the lack of cyclic changes on vaginal cytology. Ovarian transplant recipients that failed to display evidence of gonadal input postoperatively based on vaginal cytology were also excluded from analysis. Gonadal input was determined by vaginal cytology analysis, as described in *Surgical Procedures*. Mice that displayed no cyclic activity on vaginal cytology for a 10-day period before and/or after surgery were determined to have no gonadal input for said period. Mice that displayed at least one full estrous cycle in a 10-day period before and/or after surgery were determined to have gonadal input for said period. Mice that fit these criteria were the only mice used for analysis throughout this study.

### Sample collection

Mice in all groups were submitted for necropsy at death (natural death or euthanasia). Aged, moribund mice found with overt clinical signs were euthanized by inhalation of carbon dioxide. At death, all mice had major body cavities opened and were placed in 10% neutral buffered formalin for 24 hours. Subsequently, a gross necropsy was performed on each mouse and tissue collection was performed. Samples from at least 20 organ tissue categories, including, but not limited to, brain, liver, spleen, kidneys, pancreas, heart, lungs, thymus, lymph nodes, distal reproductive tract region, urinary bladder, stomach, cecum, small intestine, colon, pituitary gland, adrenal glands, ovaries, uterus, and mammary gland were examined in each mouse microscopically. Supplementary histology was conducted on any additional tissues with significant findings (in addition to those listed).

### Micro-computed tomography

Micro-computed tomography-based morphometric traits in the proximal tibia and the distal femur were measured separately for the four epiphyseal quadrants; medial femoral condyle, lateral femoral condyle, medial tibial plateau and lateral tibial plateau. Measurements were also taken for femoral and tibial mid-shaft diaphyseal cortical bone. Micro-computed tomography analysis of the selected treatment groups was performed on an Inveon Trimodality PET/SPECT/CT scanner (Siemens Preclinical Solutions, Knoxville, TN). Images consisting of 360° and 900 projections were acquired. Exposure time was 3.2 seconds with detector settings at 80 kVp and 100 μA. Data was reconstructed onto a 1600×1600×2048 image matrix using the COBRA software package (Exxim Computing Corporation, Pleasanton, CA). The effective image voxel size was 9.76 μm isotropic. Reconstructed images were analyzed and visualized using Inveon Research Workplace (4.0). A set of 3 hydroxyapatite (HA) phantoms (750 mg/ccm, 250 mg/ccm, 50 mg/ccm) were scanned and used for calibration and to compute mean density (MD).

Segmentation of the acquired images was done manually in all four epiphyseal bone quadrants. From the image slice with the largest cortical bone diameter in the subchondral bone (exclusive of osteophytes), three slices above and below or until the subchondral bone or epiphyseal plate were encountered, were segmented. In the current experiments, the entire proximal tibia and the distal femur were scanned en mass and, due to the extreme degree of mineralization in these joints, all four epiphyseal quadrants could not be aligned to optimize the volume of an ROI for each separate quadrant. In addition, severe remodeling in many joints further reduced the potential ROI for each quadrant and limited the number of slices available for analysis. The segmentation split each bone into three components: trabecular bone, cortical bone and bone marrow. After segmentation, a trabecular bone analysis tool from the IRW 3D Visualization and Analysis package was used to characterize and quantify bone morphometry.

The Inveon scanner's software utilizes a flood-fill algorithm for determining thresholds. A point was selected in the region of interest (i.e., cortical bone) and an initial default threshold was determined, which was centered over the intensity of that point. The software looked for all voxels within that threshold that were connected to it and others that were not connected (but were very likely part of the region of interest). The range was then finely adjusted to include or exclude voxels until a suitable region was selected.

The epiphyseal trabecular total volume (TV, mm^3^), trabecular bone volume (BV, mm^3^), trabecular bone volume fraction (BV/TV, %), mean trabecular thickness (Tb.Th, μm), trabecular separation (Tb.Sp, mm), trabecular number (Tb.N, 1/mm) and trabecular bone pattern factor (Tb.Pf, 1/mm) were determined. Trabecular bone pattern factor, an index of connectivity, where higher values represent a more fragmented lattice, is significantly increased in postreproductive women and is normally increased with age in female mice [22]. Tissue mineral density (TMD) was also determined. Tissue mineral density differs from BMD in that TMD is calculated from the average attenuation value of the bone tissue only and does not include attenuation values from non-bone voxels, as is done for BMD (whether volumetric or areal). Tissue mineral density measurements included MD of BV (mgHA/cm^3^) for trabecular (MD-Tb) and cortical bone (MD-Cb) and diaphyseal TMD (BD).

In mice, measurements of individual trabeculae are occasionally subject to misapproximation, due to the partial volume effect, producing a potential overestimation of trabecular thickness and a potential underestimation of trabecular TMD. Any potential overestimation of trabecular thickness or underestimation of trabecular TMD would be conducted/applied uniformly between images and groups and should not significantly influence comparisons between treatment groups.

Morphological traits of the mid-diaphyseal cortical shell were performed at a site starting approximately 9 mm distal to the proximal tibial plateau and 7 mm proximal to the distal femoral condyles and extending distal and proximal, respectively from these positions. Sequential slices were segmented and density measurements were taken. The diameter of the mid-shaft cortical bone thickness was measured using the IRW distance tool. Measurements included mid-shaft tibial bone density (BD), bone diameter (Dia) and mean thickness (MTh) and mid-shaft femoral BD, Dia and MTh. Diaphyseal bone diameter values were derived using the mean value of four, evenly spaced concentric measurements of the bone diameter.

**Fig 2:**
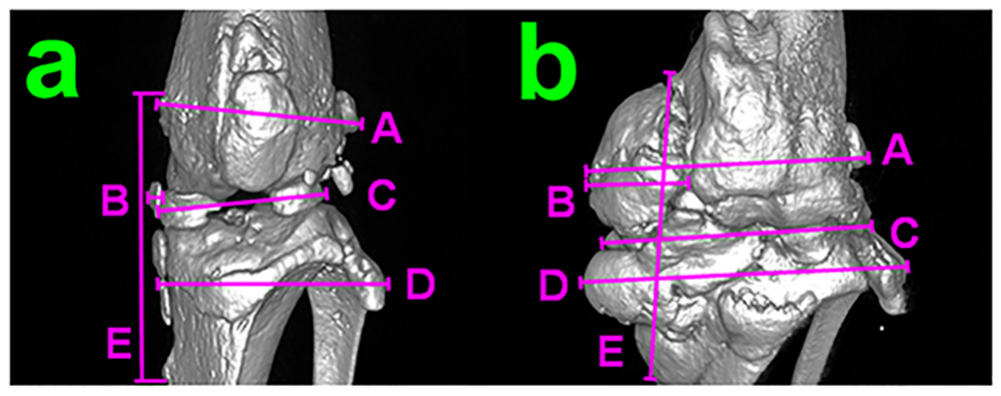
Joint grading. a) cranial view of a 3D reconstruction of uCT data from a joint with minimal perturbations. b) cranial view of a 3D reconstruction of uCT data from a joint with maximal perturbations. Measured parameters include A-distal femur width, B-medial collateral ligament thickness, C-joint width at the articulating surface, D-proximal tibia/fibula width, E-medial collateral ligament length.

For grading osteophytosis, high-resolution three-dimensional reconstructions were created. Cranial, caudal, lateral and medial images were saved and sent for analysis (Dr. Holly Mason). Analysis of the severity of osteophytosis was inclusive of syndesmophytes, enthesophytes and osteochondrophytes, with significant overlap between the diarthrodial joint pathologies. Measurements taken from the reconstructed cranial image (Fig 2) were as follows: A = width at the distal femur, B = width of medial collateral ligament (MC), C = width at the articulating surfaces, D = width at the proximal tibia/fibula, E = length of the MC. Isolated osteophytes were rare, and therefore, scores are reported as degree of joint ossification, including completeness of osteophytic joint bridging. An additional parameter; the overall width of mineralized tissue at the articulating surface (C), normalized to mid-shaft femoral diameter was relatively constant between treatments.

### Statistical analysis

Statistical analysis was performed using JMP IN 5.1 (SAS Institute Inc., Cary, NC). A Shapiro-Wilk test was used to determine normality. Data were analyzed with two-factor ANOVA and a Tukey's post-hoc test was used to determine difference between groups. Individual treatments were further analyzed by paired Student’s t-test, two-tailed, unequal distribution of variance assumed. Test results were considered significant for P values P<0.05.

## Results

The quantitative data are presented as absolute values (Tables 1, 2 and 3, S1–S4 Tables), and as percentage difference between treated groups and the IT-S mice (S1 and S2 Figs). The site of the most severe osteoarthritic changes in the stifle joint in mouse models is often reported as being in the medial tibial plateau. Due to the extreme age of many of the mice in the current study, bone changes within the stifle joint were often exaggerated to the point where changes at the medial tibial plateau appeared to have reached a 'threshold' level of severity, making it difficult to distinguish any changes between treatment groups at this single site. Therefore, results for epiphyseal bone will be reported as an average of all four epiphyseal quadrants. Differences identified between quadrants are as follows; TV, BV, BV/TV and Tb.N were slightly increased and Tb.Sp was slightly decreased from lateral to medial and from femoral to tibial compartments (S1–S4 Tables). Exceptions or trends specific to a quadrant or region are noted where appropriate.

**Table 1:** Effect on epiphyseal medial and lateral tibial plateaus and femoral condyles combined (mean values +/- SD).

*Treatment group*	*IT-S*	*IT-TX-c*	*IT-TX-a*	*OX-S*	*OX-TX*
***BV (mm*** ^***3***^ ***)***	*0*.*159 ± 0*.*061* ^a^	*0*.*128 ± 0*.*032* ^a^	*0*.*151 ± 0*.*055* ^a^	*0*.*233 ± 0*.*083* ^b^	*0*.*227 ± 0*.*079* ^b^
***TV (mm*** ^***3***^ ***)***	*2*.*29 ± 0*.*919* ^a^	*1*.*79 ± 0*.*399* ^a^	*2*.*20 ± 0*.*776* ^a^	*3*.*28 ± 0*.*843* ^b^	*2*.*98 ± 0*.*936* ^b^
***BV/TV (%)***	*0*.*072 ± 0*.*008* ^a^	*0*.*072 ± 0*.*009*	*0*.*067 ± 0*.*009* ^b^	*0*.*072 ± 0*.*012*	*0*.*076 ± 0*.*009* ^a^^,^^c^
***TbTh (mm)***	*0*.*043 ± 0*.*008* ^a^	*0*.*043 ± 0*.*006* ^a^^,^^d^	*0*.*038 ± 0*.*007* ^b^	*0*.*045 ± 0*.*009* ^a^^,^^e^	*0*.*052 ± 0*.*010* ^c^
***TbN (mm*** ^***-1***^ ***)***	*9*.*03 ± 2*.*97*	*9*.*77 ± 3*.*94*	*8*.*32 ± 2*.*82*	*8*.*07 ± 2*.*83*	*9*.*32 ± 3*.*40*
***TbSp (mm)***	*0*.*077 ± 0*.*048*	*0*.*072 ± 0*.*037*	*0*.*082 ± 0*.*039* ^a^	*0*.*082 ± 0*.*051* ^a^^,^^b^	*0*.*061 ± 0*.*028* ^c^
***Tb*.*Pf (mm*** ^***-1***^ ***)***	*11*.*2 ± 3*.*61* ^a^	*11*.*0 ± 5*.*10* ^a^	*11*.*0 ± 5*.*83* ^a^^,^^c^	*7*.*78 ± 5*.*88* ^b^^,^^c^	*7*.*13 ± 4*.*63* ^b^
***MD-Tb (mgHA/cm*** ^***3***^ ***)***	*306 ± 71*.*7* ^a^	*335 ± 67*.*6* ^a^	*280 ± 93*.*3*	*258 ± 49*.*5* ^b^	*259 ± 75*.*4* ^b^
***MD-Cb (mgHA/cm*** ^***3***^ ***)***	*342 ± 65*.*4* ^a^	*374 ±54*.*5* ^a^^,^^b^	*330 ± 71*.*8* ^a^^,^^c^	*342 ± 43*.*6* ^c^	*371 ± 44*.*9* ^b^

^a,b,c,d,e^Different alphabetic superscripts signify values significantly different from one another.

**Table 2:** Effect on diaphyseal mid-shaft tibia and femur bone parameters between treatments (mean values +/- SD).

*Mid-shaft*		*IT-S*	*IT-TX-c*	*IT-TX-a*	*OX-S*	*OX-TX*
***Tibia***	***BD (mgHA/cm*** ^***3***^ ***)***	*457 ± 35*.*7*	*483 ± 45*.*7*	*529 ± 83*.*5*	*483 ± 52*.*5*	*503 ± 89*.*0*
	***Dia (mm)***	*1*.*28 ± 0*.*058* ^a^	*1*.*38 ± 0*.*021* ^b^	*1*.*34 ± 0*.*061* ^b^	*1*.*31 ± 0*.*056*	*1*.*26 ± 0*.*074* ^a^
	***MTh (mm)***	*0*.*229 ± 0*.*012* ^a^	*0*.*217 ± 0*.*040*	*0*.*190 ± 0*.*022*	*0*.*208 ± 0*.*042*	*0*.*202 ± 0*.*033* ^b^
***Femur***	***BD (mgHA/cm*** ^***3***^ ***)***	*636 ± 59*.*3*	*670 ± 74*.*1*	*628 ± 110*	*676 ± 58*.*7*	*673 ± 54*.*3*
	***Dia (mm)***	*1*.*90 ± 0*.*140* ^a^	*1*.*88 ± 0*.*252*	*1*.*82 ± 0*.*143*	*1*.*75 ± 0*.*081* ^b^	*1*.*77 ± 0*.*150*
	***MTh (mm)***	*0*.*390 ± 0*.*174*	*0*.*362 ± 0*.*207*	*0*.*268 ± 0*.*049*	*0*.*288 ± 0*.*093*	*0*.*296 ± 0*.*127*

^a,b,c,d,e^Different alphabetic superscripts signify values significantly different from one another.

**Table 3:** Joint grading from micro-computed tomography 3D reconstructed images (mean values +/- SD).

*Treatment group*	*IT-S*	*IT-TX-c*	*IT-TX-a*	*OX-S*	*OX-TX*
***Distal femur width (mm)***	*3*.*98 ± 0*.*448* ^a^	*3*.*92 ± 0*.*289*	*3*.*64 ± 0*.*378*	*3*.*53 ± 0*.*343* ^b^	*3*.*95 ± 0*.*537*
***Width at joint space (mm)***	*3*.*65 ± 0*.*626*	*3*.*50 ± 0*.*661*	*3*.*29 ± 0*.*393*	*3*.*45 ± 0*.*587*	*3*.*65 ± 0*.*784*
***Proximal tibia/fibula width (mm)***	*4*.*55 ± 0*.*537* ^a^	*4*.*50 ± 0*.*250*	*4*.*07 ± 0*.*238* ^b^	*4*.*25 ± 0*.*577*	*4*.*35 ± 0*.*543*
***MC ossification complete***	*0*.*900 ± 0*.*376* ^a^	*0*.*833 ± 0*.*144* ^a^	*0*.*857 ± 0*.*244* ^a^	*0*.*350 ± 0*.*394* ^b^	*0*.*600 ± 0*.*555*
***MC thickness (mm)***	*0*.*972 ± 0*.*341* ^a^	*0*.*778 ± 0*.*331*	*0*.*679 ± 0*.*345*	*0*.*550 ± 0*.*284* ^b^	*0*.*861 ± 0*.*626*
***MC length (mm)***	*4*.*93 ± 0*.*426*	*5*.*33 ± 0*.*289*	*4*.*61 ± 0*.*748* ^a^	*5*.*20 ± 0*.*405*	*5*.*38 ± 0*.*626* ^b^
***LC ossicfication complete***	*0*.*600 ± 0*.*516* ^a^	*0*.*333 ± 0*.*577*	*0*.*143 ± 0*.*378*	*0*.*000 ± 0*.*000* ^b^	*0*.*250 ± 0*.*425*
***Complete med-lat ossification***	*0*.*600 ± 0*.*516* ^a^	*0*.*333 ± 0*.*577*	*0*.*000 ± 0*.*000* ^b^	*0*.*100 ± 0*.*316* ^b^	*0*.*300 ± 0*.*483*
***Joint width/mid-shaft femur dia***	*1*.*98 ± 0*.*346*	*1*.*90 ± 0*.*546*	*1*.*81 ± 0*.*185*	*2*.*03 ± 1*.*13*	*2*.*05 ± 0*.*351*
***Osteophytosis score***	*3*.*25 ± 0*.*565* ^a^	*3*.*58 ± 0*.*520* ^a^	*2*.*86 ± 0*.*690*	*2*.*01 ± 0*.*331* ^b^	*2*.*35 ± 1*.*06* ^b^

MC = medial collateral ligament; LC = lateral collateral ligament.

For MC ossification complete, LC ossification complete and Complete med-lat ossification measurements; 0 = no/none and 1 = yes/complete.

Osteophytosis score; 1 = no pathology, 2 = mild pathology, 3 = extensive conjoined, >3 surfaces, 4 = complete loss of joint architecture.

^a,b,c,d,e^Different alphabetic superscripts signify values significantly different from one another.

### Changes in epiphyseal bone

Significant changes in the micro-structure of subchondral bone were observed. Changes were similar in all four quadrants and are reported as an average value +/- SD for all quadrants (Table 1).

### Changes in diaphyseal bone

In mid-shaft diaphyseal cortical bone, a greater number of significant changes occurred in the tibia, compared to the femur. Trends for treatment effects on cortical bone were similar in the tibia and femur for MTh, but not for BD or Dia (Table 2).

### Changes in osteophytosis

Osteophytosis changes are reported as quantitative measurements and as subjective scores (1 = no pathology, 2 = mild pathology, 3 = extensive conjoined, >3 surfaces, 4 = complete loss of joint architecture) from reconstructed uCT 3D images. Osteophytosis in the stifle joints of these extreme-age mice appeared similar to ankylosis, rather than what is normally reported in osteoarthritis models. When considering the subjective osteophytosis scores, along with the quantitative measures of joint mineralization, osteophytosis was greatest in the control, IT-S (Table 3).

In some parameters, large changes occurred between groups that did not reach statistical significance. Supplemental figures (S1 and S2 Figs) are included to provide the magnitude and direction of change for each treatment and were not subject to statistical analysis. This supplemental data is presented as % change between the means of the IT-S group and the means of the OX-S sham mice and the IT-TX-c, IT-TX-a and OX-TX transplant mice for each parameter measured.

## Discussion

In addition to providing mechanical and protective function, bone also serves as a site for regulation of mineral homeostasis. This function is of particular importance in young, reproductively competent females who may need to mineralize a developing fetal skeleton or provide milk for offspring. During lactation, up to 10% of bone mass is lost in response to milk production [23]. These needs are reflected in differential regulation of bone physiology between males and females [24]. Premenopausal women have a higher cortical BMD, compared to their male counterparts [25]. This excess bone is largely endocortical, a location consistent with it being a storage site for bone that can be mobilized as required during gestation and lactation.

Bone characteristics change with age. In women, there is an increase in the rate of bone remodeling at menopause. The volume of bone resorbed increases and the volume of bone formed decreases [26]. The age-associated loss of trabecular bone in women is due to a decrease in connectivity, whereas in men, the loss is predominantly due to thinning of the individual trabecular struts [27]. The importance of mobilizing minerals in females' declines with age and bone physiology is significantly influenced by age-associated changes in ovarian function. We used observations from a previous study in this report to provide context for age-associated changes in μCT parameters in female mice. This previously-collected data was collected from wild-type female C57/BL6 mice at 8 and 20 months of age (unpublished observations, Dr. Kurt Hankenson). In the current study, we manipulated ovarian function to test four theories of ovarian influence.

First, we prepubertally OVX mice to remove all ovarian input throughout the life span of this cohort. This experiment revealed how removing all ovarian input throughout the life span would influence age-associated joint pathologies in female mice. Surprisingly, this treatment produced results vastly different from OVX in adult rodents and short-term OVX. Most experiments involving OVX are relatively short-term, and use young adult animals. Ovariectomy can have a dramatic influence on mammalian physiology immediately after gonad removal. Ovariectomized animals go through a period of adjustment and, after that period of adjustment, reach a new set-point level of physiological regulation. Ovariectomized adult rats loose trabecular BMD for about 100 days post-OVX. After 100 days, the loss of trabecular BMD stops and they maintain trabecular bone volume (albeit at a lower level) from that point forward [28]. In contrast, rats that are OVX at weaning displayed decreased bone mineralization, but greater responsiveness to exercise-induced bone remodeling [29]. This suggests that E2 participates in accumulation and maintenance of bone mineralization, but dampens the bone's response to mechanical stress. The removal of estrogenic influence in OX-S mice may have increased the responsiveness of bone to mechanical stress and may be related to the increased TV of trabecular bone demonstrated by these mice. Prepubertal OVX also appeared to exacerbate some of the negative age-associated effects in trabecular architecture and mineralization, but these mice displayed significantly better trabecular connectivity and much lower levels of osteophytosis than any other group. These results suggest that life-long lack of ovarian function significantly influences trabecular bone volume, architecture and mineralization and the level of osteophytosis.

Secondly, in OX-TX mice, we transplanted young, active ovaries to old mice that were prepubertally OVX. This experiment revealed the influence of active ovarian signaling in old mice that had no ovarian influence during development or the first half of their life span, effectively separating the influence of active ovarian signaling early in the life span from signaling late in the life span. Trabecular architecture was significantly improved in OX-TX mice, compared with all other groups. However, mineralization was not improved in trabecular bone, but was significantly increased in cortical bone. This offers strong support for the positive influence of ovarian signaling on trabecular architecture, but not mineralization late in life. These results also reveal a significant difference between the influences on mineralization in cortical versus trabecular bone.

Thirdly, in IT-TX-c mice, we transplanted young, active ovaries to old mice that were still reproductively cycling, so that reproductive function would be extended with no interruption in ovarian signaling. This experiment tested if delaying reproductive senescence would delay age-associated pathologies in female mice. This treatment appeared to delay the age-associated loss of trabecular and cortical mineral density and mildly improved trabecular architecture. Bone volume and TV were reduced in IT-TX-c mice, compared with all other groups. Both BV and TV were also non-significantly decreased by new ovaries in OVX mice, suggesting that active ovarian signaling late in the life span decreased (or prevented an increase) in BV and TV. This influence appeared to be greater on TV than on BV. Trabecular bone MD was improved in IT-TX-c mice, but not in OX-TX mice, compared with all other groups, suggesting that ovarian influence early in the life span is critical for establishment of MD-Tb, but that ovarian influence may also participate in the maintenance of MD later in life. Trabecular connectivity (Tb.Pf) was deteriorated in IT-TX-c mice, compared with the OT-TX group, but was no different from IT-S mice, suggesting that Tb.Pf is strongly influenced by ovarian signaling early in life, but may not be responsive to ovarian signaling late in life.

Lastly, in IT-TX-a mice, we transplanted young, active ovaries to old mice that had ceased reproductively cycling, so that reproductive function would be extended, but ovarian signaling would be interrupted, in direct contrast with the IT-TX-c cohort. This experiment tested if an interruption in ovarian signaling would change the influence of the newly transplanted ovaries. The basis for the existence of the 'critical period' is hypothesized to result from the loss of ERs, resulting from long-term hormone deprivation [30]. In the brain, long-term E2 deprivation leads to loss of ERs [31]. In the current experiment, our IT-TX-a mice displayed a significantly different response to the new ovaries than cycling and OVX recipients and were very different from OX-S mice as well. Acyclic recipients appeared to exacerbate the negative age-associated effects in trabecular bone seen in IT-S mice and displayed the thinnest diaphyseal bone of all cohorts. They were also the only mice to display a decrease in BV/TV, compared with IT-S mice. However, this group displayed improved osteophytosis scores, again suggesting an inverse relationship between maintenance of bone architecture and degree of osteophytosis. In a previous report, female ER-/- mice (ERα and ERβ double knockout) displayed a profound decrease in trabecular BV/TV [32], supporting the presumptive decrease in ERs in our acyclic recipients. Increased circulating levels of follicle stimulating hormone (FSH) have been linked to increased bone loss in perimenopausal women [33]. Cessation of reproductive cycling normally leads to a large increase in circulating FSH levels. In our acyclic recipients, presumptive decreases in ERs could also have decreased the negative feedback effects of E2 on FSH production, which may have led to increased FSH and the decrease in MD-Tb in these mice. A reduction in ER expression after E2 withdrawal results in a decreased response to loading in bone [34,35], which may have been the case in our acyclic recipients. The age-related loss of cortical bone, but not loss of trabecular bone can be prevented by maintaining constant E2 levels over life [36]. In rats, the relative levels of ERs are lower in cortical than cancellous bone [37,38]. This fact may have played a role in the differences seen between trabecular and cortical bone in our experiments, particularly in acyclic recipients.

## Conclusions

In summary, prepubertal OVX had the greatest influence on bone volume, whereas ovarian transplantation had the greatest effect on bone architecture. These effects may have been dictated by the timing of the treatments, with OVX appearing to influence early development and ovarian transplantation limited to influencing only the postreproductive period. However, we must also consider the physiological status or 'environment' of the mice that received new ovaries and how these different environments affected/limited the subsequent influence of the new ovaries. Responses between IT-TX-c mice and OX-TX mice that were interpreted as differences between early and late life span responses, may also be attributable to changes between the influence of the new ovary in different recipients, as was evidenced by the large differences between IT-TX-c mice and IT-TX-a mice. Major differences observed between IT-TX-c and IT-TX-a recipients may have been, in part due to differences in the levels of hormone receptors present and the responsiveness of specific bone processes to hormone signaling. Cycling recipients may have maintained ERs that were down-regulated in acyclic mice due to the gap in E2 exposure prior to ovarian transplantation. If the IT-TX-a mice were, in fact less responsive to the new ovaries than cycling mice and displayed improved osteophytosis scores, then this supports the view that osteophytosis is a compensatory response to changes within or acting upon the joint.

Transplantation of new ovaries had little influence on diaphyseal bone compared with trabecular bone. Trabecular bone has more surface per unit bone volume than cortical bone, so that trabecular bone is more likely to be remodeled than cortical bone [39]. In the current experiments, transplantation-induced changes necessarily took place after 11 months of age, suggesting that these parameters are more amenable to changes later in life. This suggestion is supported by a similar pattern of changes occurring in IT-TX mice that received new ovaries at 11 months of age. Overall, cortical mineralization and osteophytosis were increased by ovarian transplantation in cycling and OVX mice, but decreased by new ovaries in acyclic mice and by prepubertal OVX. In OVX adult female macaques, long-term HRT had no significant effect on cross-sectional area of osteophytes [40]. In our experiments, the presence of a senescent ovary was consistent with a high level of osteophytosis, which was reduced in OVX mice and not significantly increased in mice with new ovaries. These observations suggest a potentially causative role for signaling from the senescent ovary in the development of osteophytosis in normal, aged IT-S mice.

Our perceived treatment changes, reported as increases or decreases compared with IT-S mice may alternatively may have been just the maintenance of a level bone regulation previously present at a younger age. Future work will include dissecting the role of hormone receptors and the role of the senescent ovary in orthopedic disease progression. These findings may suggest that tailoring HRT therapy to the patient's current reproductive status may improve therapy effectiveness.

## Supporting Information

S1 Fig Percent difference (treatment/IT-S) between intact sham mice and all other treatments for BV, TV, BV/TV, Tb.Th, Tb.N, Tb.Sp, MD-Tb, MD-Cb and Tb.Pf. (TIF)

S2 Fig Percent difference (treatment/IT-S) between intact sham mice and all other treatments for T-BD, T-Dia, T-MTh, F-BD, F-Dia, F-MTh, Osteophytosis Score, Joint width/femur diameter. (TIF)

S1 Table Effect on epiphyseal lateral femoral condyles (means +/- SD). (XLSX)

S2 Table Effect on epiphyseal medial femoral condyles (means +/- SD). (XLSX)

S3 Table Effect on epiphyseal lateral tibial plateau (means +/- SD). (XLSX)

S4 Table Effect on epiphyseal medial tibial plateau (means +/- SD). (XLSX)
