# Supplementary figures and images for: Manipulation of Ovarian Function Significantly Influenced Trabecular and Cortical Bone Volume, Architecture and Density in Mice at Death
Source: PLoS One. 2015 Dec 30;10(12):e0145821. doi: 10.1371/journal.pone.0145821 (PMC4696788; doi:10.1371/journal.pone.0145821)

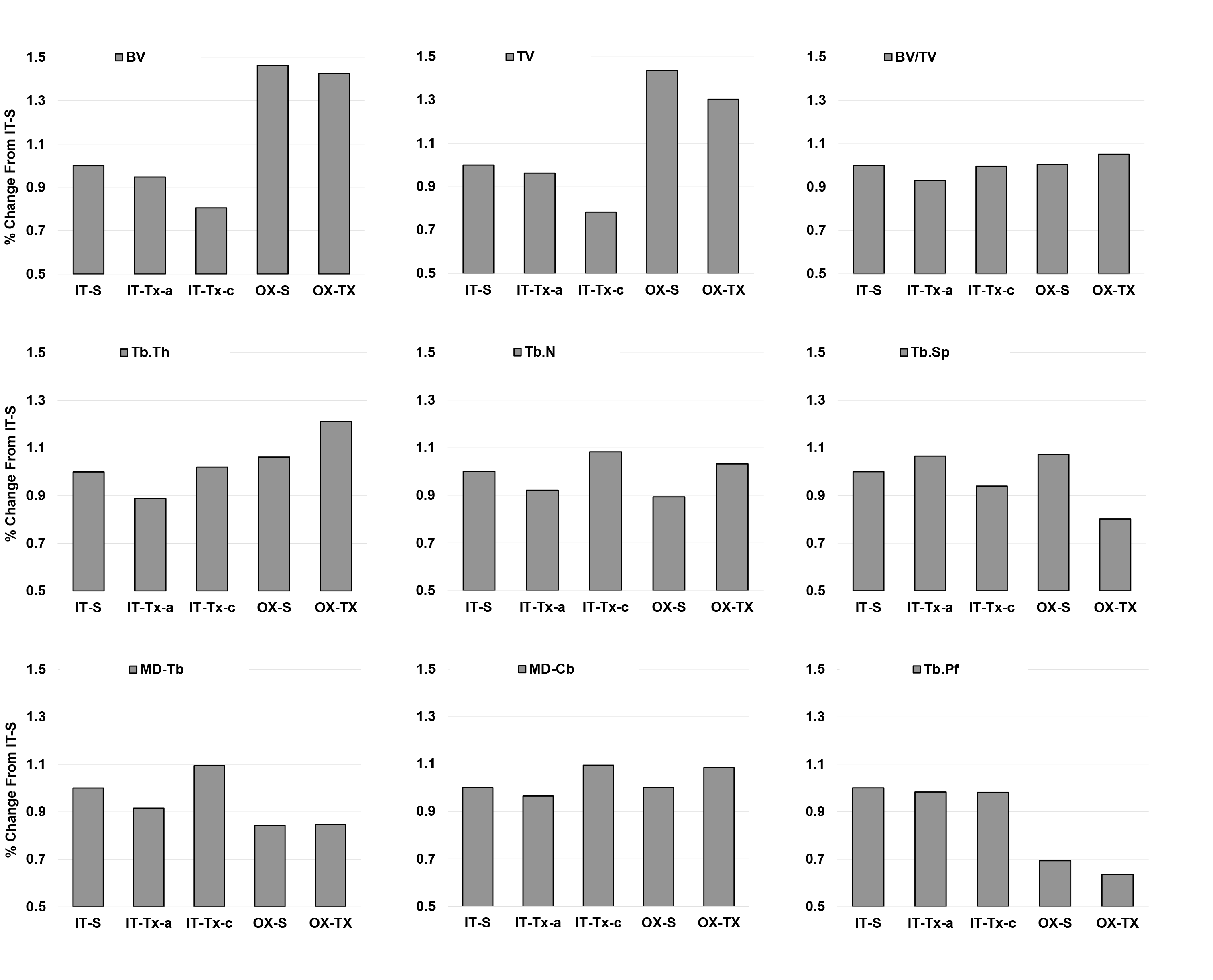

Supplement: S1 Fig — (TIF) [file pone.0145821.s001.tif]

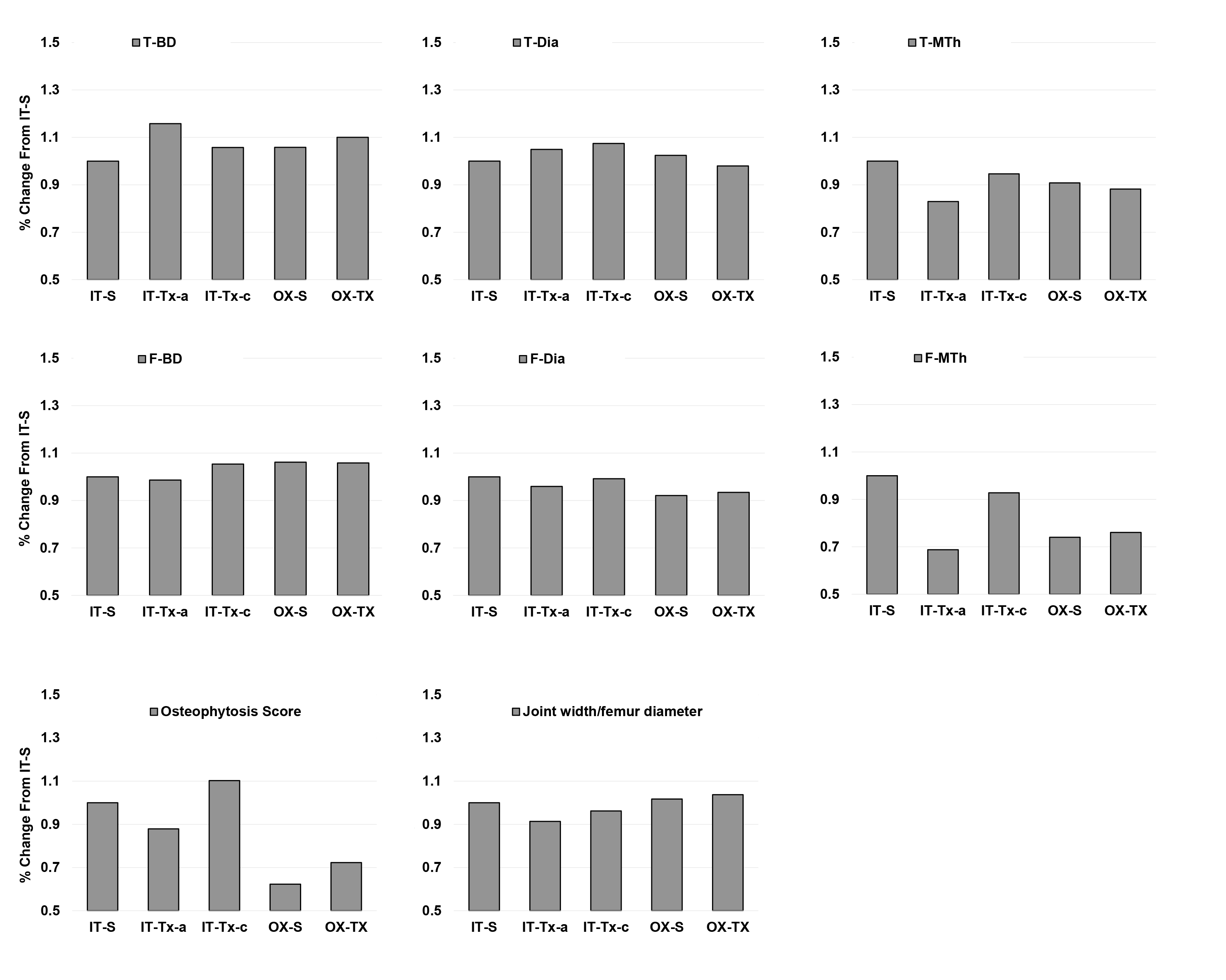

Supplement: S2 Fig — (TIF) [file pone.0145821.s002.tif]
